# The alteration of bile acids and gut microbiota is associated with intestinal barrier dysfunction and inflammaging in human

**DOI:** 10.3389/fragi.2026.1741360

**Published:** 2026-04-15

**Authors:** Yumei Xu, Liang Zhang, Tianci Li, Yanqiu Zhao, Shuo Wang, Junwen Qi, Linsen Shi

**Affiliations:** 1 Department of Radiation, The Fourth Affiliated Hospital of Soochow University, Suzhou, Jiangsu, China; 2 Department of Gastrointestinal Surgery, Xuzhou Central Hospital, Xuzhou Clinical School of Xuzhou Medical University, Xuzhou, Jiangsu, China; 3 Affiliated First Clinical College, Xuzhou Medical University, Xuzhou, Jiangsu, China; 4 Department of General Surgery, The Fourth Affiliated Hospital of Soochow University, Suzhou, Jiangsu, China; 5 Medical Center of Soochow University, Soochow University, Suzhou, Jiangsu, China

**Keywords:** aging, bile acids, gut microbiota, inflammation, intestinal barrier

## Abstract

**Background:**

The increasing prevalence of age-related chronic diseases, driven by the aging population, poses substantial medical and economic challenges. Emerging researches have underscored the crucial roles of gut microbiota and bile acids (BAs) in metabolic and physiological functions regulation.

**Methods:**

100 elderly and 100 young participants were enrolled in this study. Fecal and serum BAs were quantified by liquid chromatography-tandem mass spectrometry (LC-MS/MS), while gut microbiota composition was assessed through 16S rRNA gene sequencing. Cytokine levels were measured by Enzyme-Linked Immunosorbent Assay (ELISA).

**Results:**

Elderly participants exhibited significantly lower levels of primary fecal BAs, particularly cholic acid (CA) and chenodeoxycholic acid (CDCA), alongside an increase in secondary BAs such as lithocholic acid (LCA), leading to a marked reduction in the primary/secondary BAs ratio. Serum showed a decline in both conjugated and unconjugated BAs, primary/secondary BAs ratio, while a notable rise in 12α-OH/non-12α-OH BAs. Furthermore, increased levels of P21, LPS, IL-6, and TNF-α in the elderly were associated with specific BA changes, including reduced fecal unconjugated primary BAs and increased LCA. Significant differences in gut microbiota composition were observed, with the elderly displaying a higher abundance of microbiota capable of 7α-dehydroxylation. Correlations were observed among BAs, gut microbiota alterations, and markers of chronic inflammation and intestinal barrier dysfunction.

**Conclusion:**

Aging is associated with significant changes in the BA pool, which are associated with gut microbiota dysbiosis. These alterations may be related to intestinal barrier dysfunction and chronic low-grade inflammation. Modulating BA metabolism presents a potential strategy for mitigating the aging process. Due to the cross-sectional design, causal relationships cannot be established.

## Background

With significant advancements in medical technology and public healthcare, aging is becoming a pressing social issue. By 2050, the global elderly population is projected to surpass 1.6 billion, marking the onset of an era of profound demographic aging (World Population Prospects, 2022). Consequently, research about senescence and related diseases has gained more and more attention. Aging, a natural and irreversible physiological process, involves the continuous degeneration and decline of cells, tissues, organs, and the entire organism (Jang et al., 2024). It typically results from the intricate interplay of genetics, environmental, and lifestyle factors, leading to the gradual functional and structural loss of biological molecules, cells, and tissues (Bana and Cabreiro, 2019). Studies have explored aging characteristics and underlying mechanisms, linking aging to factors like genomic instability, telomere attrition, epigenetic alterations, proteostasis loss, impaired autophagy, dysregulated nutrient sensing, mitochondrial dysfunction, cellular senescence, stem cell exhaustion, altered intercellular communication, chronic inflammation, and ecological imbalance (López-Otín et al., 2023).

Gut microbiota, often referred to as the body’s “second genome,” plays an essential roles in metabolism, immune response, and gene expression (Tropini et al., 2017). Gut dysbiosis is implicated in various aging related disorders, such as digestive, metabolic, cardiovascular, and neurological diseases (Vespasiani -Gentilucci et al., 2018; Hill and Round, 2021). Bile acids (BAs), byproducts of cholesterol metabolism in the liver, are not only vital for lipid digestion and absorption of lipid-soluble vitamins but also act as signaling molecules influencing aging, inflammation control, immune homeostasis, and tumor development (Lavelle and Sokol, 2020; Campbell et al., 2020). They regulate gut microbiota growth and composition, while gut microbiota significantly influence BA hydrolysis and the synthesis of secondary and tertiary BAs (Jia et al., 2018). This interplay affects immune function and metabolic phenotypes, evently may contribute to obesity, diabetes, non-alcoholic fatty liver disease, inflammatory bowel disease, and certain cancers (Cai et al., 2022; Liu et al., 2024).

The gastrointestinal tract, beyond its roles in nutrient metabolism and immune regulation, serves as a critical defense against harmful substances entering the circulation, a function known as the intestinal barrier (Turner, 2009). Impaired intestinal barrier function is linked to various intestinal and systemic diseases (Odenwald and Turner, 2017; Paone and Cani, 2020). Both intestinal microbiota and small molecule metabolites, such as BAs, involved in the regulation of this barrier function (Camilleri, 2019; Shi et al., 2023; Untersmayr et al., 2022). Deterioration of the intestinal barrier is a key factor in systemic chronic inflammation and immune dysregulation (Salazar et al., 2023), often considered a hallmark of aging (Oishi and Manabe, 2016). Chronic inflammation, a typical pathological aspect of aging and age-related diseases (Bottazzi et al., 2026), is characterized by the natural expression and production of inflammatory mediators (Ferrucci and Fabbri, 2018). Most older adults exhibit a progressive increase in inflammatory state, marked by rising blood levels of inflammatory markers, a condition referred to as inflammaging (Fulop et al., 2026). Prolonged subclinical chronic inflammation heightens susceptibility to chronic diseases and accelerates aging (Huang et al., 2022).

Through this research,we will investigate the correlation between BAs metabolism and gut microbiota in aging by comparatively analysing BAs profiles and gut microbiota in older and younger adults. We also measured inflammatory and intestinal barrier-associated cytokines to investigate their correlation with BAs and gut microbiota. This research aims to elucidate the role of gut microbiota-mediated BA metabolism in aging. Understanding gut microbiota and BA metabolism changes during aging, and how the gut microbiota-bile acid axis influences aging, can offer new insights for preventing and treating elderly diseases, providing a reliable reference for senescence management.

## Materials and methods

### Study population

A total of 200 individuals undergoing routine health check-ups were recruited for this study at The Affiliated Hospital of Xuzhou Medical University between May 2022 and May 2023. The cohort consisted of 100 young individuals (18–35 years old) and 100 elderly individuals (≥65 years old).

“Healthy” individuals were defined as those without diagnosed chronic diseases, including cardiovascular disease, diabetes mellitus, liver disease, gastrointestinal disorders, renal dysfunction, autoimmune diseases, or active infections, and without abnormal clinical symptoms at the time of enrollment. In addition, participants were not receiving medications known to affect bile acid metabolism or gut microbiota composition.

Inclusion criteria were: (1) individuals aged 18–35 years or ≥65 years; (2) absence of diagnosed chronic diseases as defined above; and (3) willingness to participate and provide informed consent. Exclusion criteria included: (1) use of antibiotics, probiotics, bile acid-binding agents, corticosteroids, or other medications affecting gut microbiota or metabolism within the past 3 months; (2) presence of gastrointestinal symptoms such as diarrhea, constipation, or abdominal pain within the past month; and (3) any acute illness or infection prior to sampling. All participants provided written informed consent prior to inclusion. The study was conducted in accordance with the Declaration of Helsinki and was approved by the Ethics Committee of The Affiliated Hospital of Xuzhou Medical University.

### Clinical data and specimen collection

Participant data collected encompassed gender, age, height, and weight, with Body Mass Index (BMI) calculated as weight in kilograms divided by height in meters squared. Blood pressure was measured by medical professionals. Laboratory tests assessed blood lipids, glucose, aspartate aminotransferase, alanine aminotransferase, creatinine, bilirubin, among other biochemical markers.

For each participant, 5 mL of fasting venous blood from the forearm was collected into an anticoagulant-containing tube and kept at 4 °C for 1 hour for clotting. Following this, it was centrifuged at 5,000 rpm for 10 min at 4 °C. The serum, confirmed free from hemolysis, was transferred to a sterile EP tube and stored at −80 °C for subsequent BA and cytokine analysis. Protease inhibitors were not added during sample collection; however, all samples were processed under low-temperature conditions to minimize protein degradation. Simultaneously, a mid-point fecal sample (≥1 g) was collected in the morning, placed in a sterile EP tube under anaerobic conditions, flash-frozen in liquid nitrogen, and stored at −80 °C for fecal microbiota and BA analysis.

### Quantification of fecal and serum BAs

BA measurements in serum and feces utilized a triple quadrupole liquid chromatography-tandem mass spectrometry (LC-MS/MS) system (AB Sciex Triple Quad™ 5500 LC/MS/MS system). For serum samples, 100 µL of serum was taken directly for analysis. For fecal samples, 10 mg of freeze-dried material was combined with 500 μL of 70% ethanol, ultrasonicated for 30 min, and then centrifuged at 13,000 rpm for 10 min at 4 °C. 100 μL of the supernatant was taken for analysis. 100 μL of serum or the fecal extract was mixed with 600 μL of acetonitrile containing the internal standard dhCA (100 ng/mL), followed by vortexing and centrifugation to collect 600 μL of the supernatant. The supernatant was dried, concentrated, reconstituted in 100 μL of methanol, and 4 μL of this mixture was injected for analysis. Chromatographic separation occurred on an Agilent C18 column (1.8μm, 2.1 mm × 100 mm, I.D.: 758700-902) using water (5 mmol/L ammonium acetate solution) and acetonitrile in a gradient elution. BA metabolites were detected in multiple reaction monitoring mode, with quantification based on a standard curve. Analyst 1.4.2 software (AB Sciex, Foster City, CA, USA) was employed for BA detection.

### Fecal microbiota sequencing analysis

Fecal microbiota sequencing was conducted by Biomarker Technologies Co. (Beijing, China). Total DNA was extracted from samples, and primers targeting the V3-V4 region of the 16S rRNA gene were used (Primer Sequence: F: ACT​CCT​ACG​GGA​GGC​AGC​A; R: GGACTACHVGGGTWTCTAAT). Sequencing adapters were added to the primers, and the target sequences were amplified by PCR. The PCR products were then purified, quantified, and homogenized to form a sequencing library. This library was sequenced using the Illumina HiSeq 2500 system to produce raw reads. Initial data filtering based on single nucleotide quality was performed using Trimmomatic (version 0.33). Primer sequences were removed using Cutadapt (version 1.9.1). Paired-end reads were assembled using USEARCH(version 10), with chimera removal by UCHIME (version 8.1). The resulting high-quality reads were clustered into OTUs with a similarity threshold of 97.0% using USEARCH, and taxonomic annotation was conducted using a bayesian classifier with the Silva.138 database as a reference.

Subsequent analyses, including alpha and beta diversity, differential group analysis, correlation analysis, and function prediction, were performed on BMKCloud (https://international.biocloud.net).

### Enzyme‐Linked immunosorbent assay (ELISA)

Cytokines (P21, LPS, IL-6, and TNF-α) were measured using commercial ELISA kits, following the manufacturer’s instructions. Both of kits were sourced from RUIXIN BIOTECH (Quanzhou, China).

### Statistical analyses

Statistical analyses were executed with SPSS (version 27.0.1.0; IBM Corp.) and R. Normally distributed data were presented as mean ± SD, and non-normally distributed data as median (25th, 75th percentiles). Categorical data, such as gender, were analyzed using the chi-square test. Outliers beyond mean ±3 standard deviations were removed. The Shapiro-Wilk normality test and Levene’s test assessed the normal distribution and variance homogeneity, respectively. Depending on these assessments, data were analyzed using an independent samples t-test, Welch’s t-test, or the Wilcoxon rank sum test. Spearman correlation tested relationships. Significance was set at *P* < 0.05. Visualization was performed using GraphPad Prism (version 9.5.1; GraphPad Software) and ggplot2 in R.

## Results

### Clinical characteristics of the study population

The study enrolled 100 elderly and 100 young individuals. Clinical data and characteristics are detailed in Table 1. Notably, compared to the young group, the elderly group showed significantly higher levels of systolic blood pressure (SBP) (*P* < 0.001), diastolic blood pressure (DBP) (*P* < 0.001), blood glucose (GLU) (*P* < 0.001), total cholesterol (TC) (*P* = 0.002), aspartate aminotransferase (AST) (*P* = 0.045), direct bilirubin (DBIL) (*P* = 0.004), and creatinine (Cr) (*P* = 0.016). Conversely, levels of high-density lipoprotein cholesterol (HDL-C) were significantly lower in the elderly group (*P* = 0.025).

**TABLE 1:** Clinical characteristics of young and old people.

Characteristics	Yong (n = 100)	Old (n = 100)	*p* value
Age (Years)	29.000 (23.0,32.0)	73.000 (70.0,78.0)	2.36e-34***
Gender (Male: Female)	50:50	52:48	0.777
BMI (Kg/m2)	24.07 ± 3.52	23.58 ± 3.09	0.302
SBP (mmHg)	117.000 (110.3,122.0)	135.000 (129.3,140.0)	5.62e-30***
DBP (mmHg)	73.000 (70.0,78.0)	87.000 (81.3,92.0)	2.41e-23***
GLU (mmol/L)	4.810 (4.5,5.2)	5.355 (4.8,6.5)	3.48e-07***
TC (mmol/L)	4.22 ± 0.77	4.63 ± 1.04	0.002**
TG (mmol/L)	1.070 (0.8,1.4)	1.160 (0.9,1.6)	0.074
HDL-C (mmol/L)	1.180 (1.0,1.3)	1.090 (0.9,1.3)	0.025*
LDL-C (mmol/L)	2.57 ± 0.60	2.50 ± 0.81	0.483
AST (U/L)	18.000 (14.8,21.0)	19.000 (16.0,24.5)	0.045*
ALT (U/L)	16.000 (10.0,24.0)	16.000 (11.0,22.0)	0.766
TBIL (μmol/L)	9.600 (7.4,12.6)	10.700 (7.9,14.4)	0.057
DBIL (μmol/L)	3.900 (2.7,4.7)	4.600 (3.3,6.3)	0.004**
IBIL (μmol/L)	5.650 (4.4,7.8)	5.800 (4.2,8.4)	0.514
Cr (μmol/L)	56.000 (45.0,64.0)	58.500 (51.0,69.8)	0.016*

BMI, body mass index; SBP, systolic blood pressure; DBP, diastolic blood pressure; GLU: glucose; TC: total cholesterol; TG, triglycerides; HDL-C, high density lipoprotein cholesterol; LDL-C, Low-density lipoprotein cholesterol; AST, aspartate aminotransferase; ALT, alanine aminotransferase; TBIL, total bilirubin; DBIL, direct bilirubin; IBIL, indirect bilirubin; Cr: Creatinine.

Gender distribution differences were analyzed using the Chi-square test. Clinical characteristics data are presented as mean ± SD, or median (25th, 75th percentiles), based on normal distribution conformity. Mean differences were evaluated using independent samples t-test, and median differences using the Wilcoxon rank sum test. Significant p-values are indicated (*P < 0.05, **P < 0.01, ***P < 0.01).

### Alteration of BAs levels between elderly and young participants

17 BAs was tested with fecal and serum samples from 200 participants: cholic acid (CA), chenodeoxycholic acid (CDCA), ursodeoxycholic acid (UDCA), lithocholic acid (LCA), deoxycholic acid (DCA), hyodeoxycholic acid (HDCA), glycocholic acid (GCA), glycine chenodeoxycholic acid (GCDCA), glycine ursodeoxycholic acid (GUDCA), glycine lithocholic acid (GLCA), glycine deoxycholic acid (GDCA), taurocholic acid (TCA), taurochenodeoxycholic acid (TCDCA), tauroursodeoxycholic acid (TUDCA), taurolithocholic acid (TLCA), taurodeoxycholic acid (TDCA), and taurohyodeoxycholic acid (THDCA). The serum level of THDCA was too low for statistical inclusion. CA, CDCA, and their conjugated forms were classified as primary BAs, while UDCA, LCA, DCA, HDCA, and their conjugates were classified as secondary BAs. Unconjugated BAs comprised CA, CDCA, UDCA, LCA, DCA, and HDCA, the rest defined as conjugated BAs. 12α-OH BAs included CA, DCA, and their conjugated forms. Non-12α-OH BAs included CDCA, UDCA, LCA, HDCA, and their conjugated.

**FIGURE 1:**
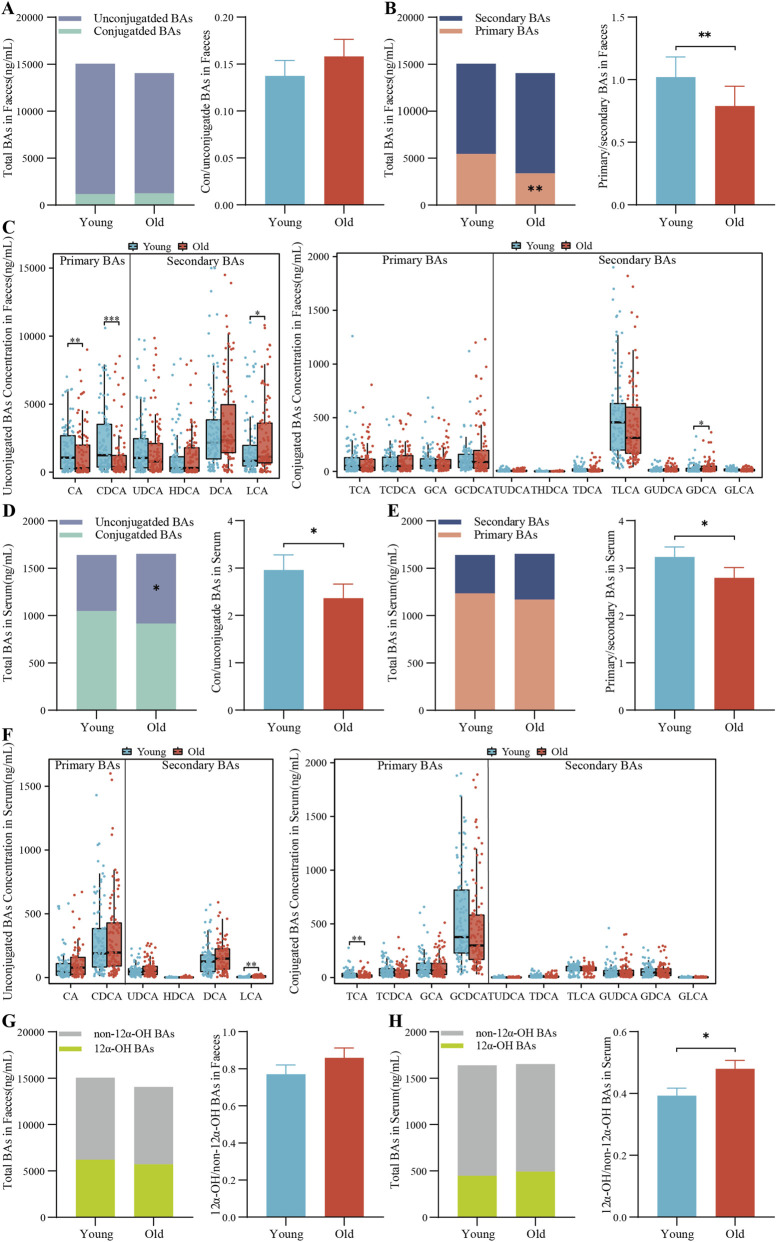
BA profile in the feces and serum of young and elderly individuals. This includes total BAs ratios of conjugated to unconjugated, and primary to secondary BAs, along with concentrations of individual BAs in feces **(A–C)** and serum **(D–F)**. Ratios of 12α-OH BAs to non-12α-OH BAs in feces **(G)** and serum **(H)** are also presented. Data are expressed as mean ± SD or median (25th, 75th percentiles), based on their distribution normality. Differences in means were analyzed using an independent samples t-test, while medians were analyzed using the Wilcoxon rank sum test. Significant p-values are indicated. (*P < 0.05, **P < 0.01, ***P < 0.01).

Fecal total BA levels did not significantly differ between older and younger participants (Figure 1A). Although not statistically significant, the fecal conjugated/unconjugated BAs ratio was higher in older participants (Figure 1A). Older participants had significantly lower levels of fecal primary BAs than younger ones (*P* = 0.001), resulting in a significantly lower primary/secondary BAs ratio (*P* = 0.007) as well (Figure 1B). No significant differences were observed in the fecal levels of 12α-OH-BAs and non-12α-OH-BAs or their ratio (Figure 1G). Individually, younger participants had significantly higher fecal levels of primary BAs: CA (*P* = 0.002) and CDCA (*p* < 0.001), whereas secondary BAs, LCA (*P* = 0.034) and GDCA (*P* = 0.020), were higher in older participants (Figure 1C).

In serum, total BA levels were not significantly different between groups. Intriguingly, the serum conjugated/unconjugated BA ratio showed an opposite trend to fecal samples. Unconjugated BA levels were significantly higher in the elderly participants (*P* = 0.033), leading to a lower conjugated/unconjugated BAs ratio compared to the young participants (*P* = 0.011) (Figure 1D). Consistent with the fecal findings, the primary/secondary BAs ratio in serum was significantly lower in the elderly (*P* = 0.043) (Figure 1E). The 12α-OH/non-12α-OH BAs ratio was significantly higher in the elderly group (*P* = 0.031) (Figure 1H). Individually, serum LCA levels were significantly elevated in the elderly (*P* = 0.006), while TCA levels were higher in young participants (*P* = 0.008) (Figure 1F).

### Aging and inflammation related cytokines increase significantly in the elderly

**FIGURE 2:**
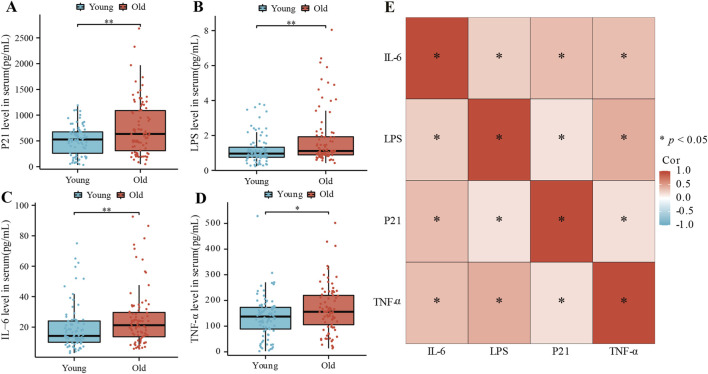
Serum cytokine levels in younger and older participants. ELISA was used to measure levels of P21 **(A)** LPS **(B)** IL-6 **(C)** and TNF-α **(D)** in serum. Data are presented as median values (25th, 75th percentiles). Significant p values are marked. **(E)** Correlation analysis among the four cytokines. (*P < 0.05, **P < 0.01, ***P < 0.01).

During aging, senescent cells often secrete certain cytokines, reflecting the body’s aging and inflammation status. A characteristic “leakage” in the intestinal barrier is a primary aging feature, associated with inflammaging and immune senescence (Salazar et al., 2023; Oishi and Manabe, 2016). Given the GI tract’s role in BA synthesis and metabolism by gut microbiota, we investigated whether alterations in BA profiles and gut microbiome during aging contribute to intestinal barrier leakage and participate in the aging process. P21, a recognized ageing biomarker (Schoultz and Keita, 2020), and serum LPS levels, indicative of intestinal permeability (Balkwill and Mantovani, 2001), were measured. Additionally, IL-6 and TNF-α, key inflammatory factors (Gonzalez, 2012) were analyzed. ELISA measured the serum levels of P21, LPS, IL-6, and TNF-α in both groups. As indicated in Figure 2, levels of P21 (*P* = 0.008) (Figure 2A), LPS (*P* = 0.009) (Figure 2B), IL-6 (*P* = 0.003) (Figure 2C), and TNF-α (*P* = 0.019) (Figure 2D) were significantly higher in the elderly. These results suggest an impaired intestinal barrier function and increased systemic chronic inflammation in elderly individuals, leading to a fragile state. Correlation analysis (Figure 2E) revealed significant correlations among these four cytokines, indicating a potential interrelationship between aging, impaired intestinal barrier function, and inflammation.

### The clinical characteristics and cytokine levels were associated with changes in BA composition

**FIGURE 3:**
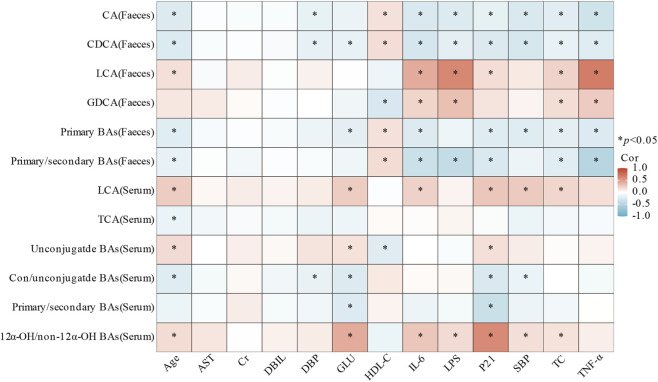
Heat map of the Spearman correlation analysis between diverse BAs and clinical characteristics (*P < 0.05).

A Spearman correlation analysis was performed to examine the association between changes in BAs composition and variations in clinical characteristics and serum cytokine levels. The findings are depicted in Figure 3. In feces, CA, CDCA, primary BAs, and the primary/secondary BAs ratio were strongly negatively correlated with age, BP, GLU, TC, and cytokines, while positively correlated with HDL-C. Conversely, fecal LCA, GDCA, along with serum LCA, unconjugated BAs, and the 12α-OH/non-12α-OH BAs ratio exhibited an inverse trend. Additionally, serum TCA was inversely correlated with age. Notably, both the serum primary/secondary and conjugated/unconjugated BAs ratios were negatively correlated with P21 and GLU. Furthermore, the conjugated/unconjugated BAs ratio also showed significant negative correlations with age and BP.

### Variations in gut microbiota structures between elderly and young participants

**FIGURE 4:**
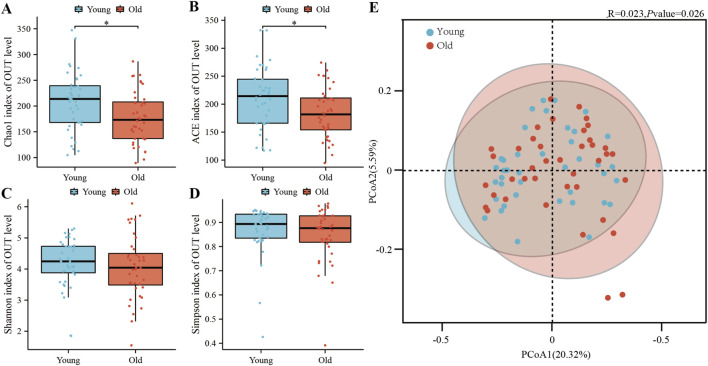
Intestinal microbial diversity analysis in two groups. Alpha diversity was assessed at the OTU level using the Chao1 index **(A)** ACE index **(B)** Shannon index **(C)** and Simpson index **(D)**. Principal coordinate analysis **(E)** was based on binary Jaccard distance. Significant *p* values are marked (*P < 0.05).

To investigate the differences in gut microbiota between elderly and young participants, and their association with BA composition, we analyzed 40 fecal samples from each group through gut microbiota sequencing. This process yielded 12,360,477 paired-end reads, which, after quality control and assembly, resulted in 12,314,663 clean reads. The Usearch software clustered these reads into 471 operational taxonomic units (OTUs), with 441 OTUs common to both groups, 27 unique to the elderly, and 3 unique to the young. Subsequent alpha and beta diversity analyses, based on these OTUs, were conducted to examine variations in microbial richness and composition. As illustrated in Figure 4, the Chao1 index (Figure 4A) and ACE index (Figure 4B), indicators of species richness, were significantly higher in the young group compared to the elderly. However, the Shannon index (Figure 4C) and Simpson index (Figure 4D), which measure species diversity, showed no notable differences between the groups. This indicates that the gut microbiota of young individuals exhibits higher species richness than that of the elderly, with no significant disparity in species diversity. Principal coordinate analysis (PCoA) (Figure 4E), based on the Binary-Jaccard distance, also revealed a distinct separation in the microbiota composition of the two groups (*p* < 0.05), signifying a significant difference in their microbiota compositions.

**FIGURE 5:**
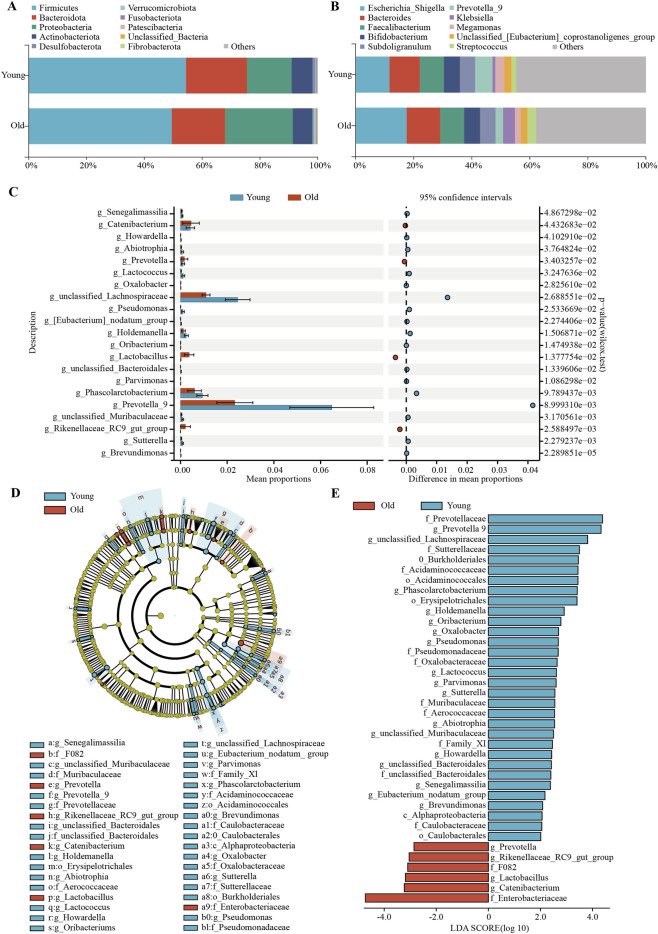
Gut microbial composition analysis. Bar plots illustrate the top 10 most abundant species at the phylum **(A)** and genus **(B)** levels based on relative abundances. **(C)** STAMP analysis identifies differential flora at the genus level. **(D)** Cladogram from LEfSe analysis. **(E)** Differences in gut microbiota between the two groups were identified using LEfSe analysis, with *p* < 0.05 and LDA score threshold >2.0.

The analysis of microbial composition differences highlighted the microbial composition across various taxonomic levels. Figure 5A displays the top 10 most abundant phyla, revealing that both groups’ gut microbiota were predominantly composed of *Firmicutes*, *Bacteroidetes*, *Proteobacteria*, and *Actinobacteriota*, collectively constituting over 95% of all phyla. Figure 5B shows the top 10 genera in terms of relative abundance. We then performed a differential analysis of all gut microbial taxa at both phylum and genus levels using the Wilcoxon rank sum test in STAMP analysis. This revealed no significant differences at the phylum level. However, at the genus level, we identified 21 significantly differentially expressed gut microbiota, as depicted in Figure 5C. Among these, *Senegalimassiliag, Howardella, Abiotrophia, Lactococcus, Oxalobacterg, unclassified_Lachnospiraceae, Pseudomonas, [Eubacterium]_nodatum_group, Holdemanella, Oribacterium, Unclassified_Bacteroidales, Parvimonas, Phascolarctobacterium, Prevotella_9, unclassified_Muribaculaceae, Sutterella,* and *Brevundimonas* were relatively more abundant in younger individuals. Conversely, *Catenibacterium, Prevotella, Lactobacillus,* and *Rikenellaceae_RC9_gut_group* were more prevalent in older individuals.

To identify differences in specific taxa between the elderly and young groups, we conducted a LEfSe analysis from the phylum to genus level, employing effect size measurements to highlight bacterial taxa with differing abundances between the groups. With the threshold of significance (*P* < 0.05) and LDA score >2, a total of 38 gut microbial taxa were identified as significant, with 32 enriched in the young group and 6 in the elderly group. This finding aligns with the results of the STAMP analysis. Taxa with varying abundances are illustrated in Figure 5E. Additionally, cladograms generated from the LEfSe analysis visually represent the phylogenetic distribution of these samples, from class to genus level, with the size of each circle in the cladogram indicating the abundance of specific taxa (Figure 5D).

### Changes in gut microbiota structure related to BA metabolism

**FIGURE 6:**
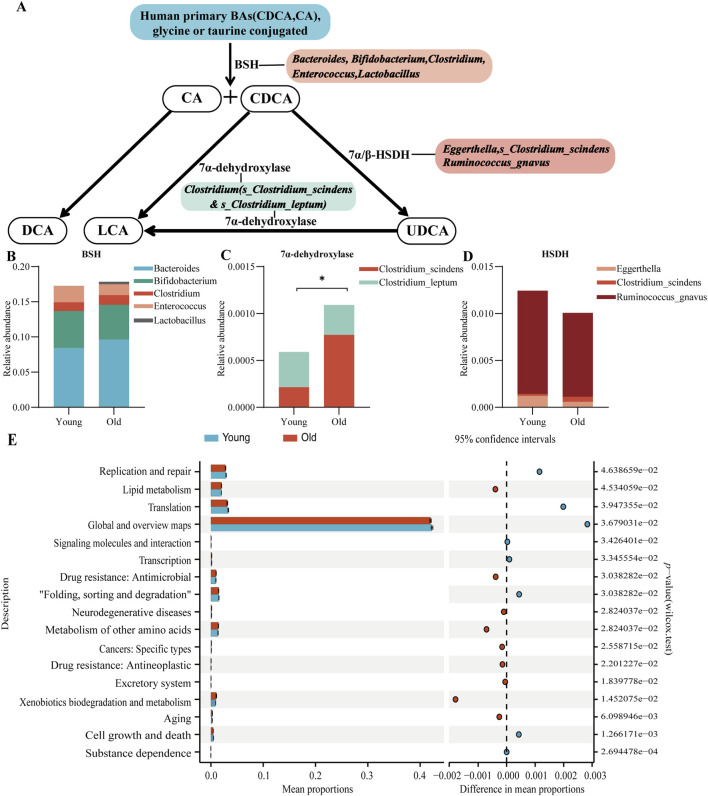
Gut microbiota related to BA metabolism and predicted functional profiles. **(A)** Main biological transformations of BAs by gut microbiota. Composition of gut microbiota related to BSH **(B)** 7α-dehydroxylase **(C)** and 7α/β-HSDH **(D)**. **(E)** Functional abundance of gut microbiota was predicted using PICRUSt 2, and differential functional analysis was conducted at the KEGG level 2 (*P < 0.05).

The intestinal microbiota is involved in BAs biotransformation, influencing and being influenced by the composition and abundance of BAs (Cai et al., 2022). Approximately 5%–10% of BAs are secreted into the colon, undergoing biotransformation by the intestinal microbiota before reabsorption or fecal excretion (Guo et al., 2022). Figure 6A illustrates that the impact of gut microbiota on BAs composition and secondary BAs synthesis includes three key processes: deconjugation, dehydroxylation, and oxidation-isomerization of BAs (Thomas et al., 2008). Bile salt hydrolase (BSH) enzymes catalyze the deconjugation of BAs, forming unconjugated BAs (CA, CDCA) (Zheng et al., 2015). Then, bacterial dehydroxylation enzymes convert CA and CDCA into secondary BAs, DCA and LCA, respectively. Additionally, CDCA can be transformed into UDCA by steroid dehydrogenase (HSDH) (Xiao et al., 2021), which can further be converted to LCA via dehydroxylation enzymes (Jia et al., 2018).

Therefore, we further analysed each of the gut microbiota found in previous studies to be associated with BSH (Foley et al., 2021), 7α-dehydroxylase (Ridlon et al., 2020), and 7α/β-HSDH (Fogelson et al., 2023) of BAs. There were no significant differences in the overall abundance of BA BSH-associated gut microbiota (*P* = 0.544) (Figure 6B), with only *Lactobacillus* (*P* = 0.014) being significantly increased in the elderly, while *Bacteroides* (*P* = 0.583)*, Bifidobacterium* (*P* = 0.525)*, Clostridium* (*P* = 0.104) and *Enterococcus* (*P* = 0.413) not being significantly different. *Clostridium_scindens* and *Clostridium_leptum* are considered to be the crucial bacterial groups for BA 7α-dehydroxylation, and our sequencing results identified these two bacterial groups at the species level. *Clostridium_scindens* showed a significant increase in older participants (*P* = 0.002), while *Clostridium_leptum* did not change significantly (*P* = 0.292). The overall abundance of the two aforementioned flora was also significantly higher (*P* = 0.013) in older participants (Figure 6C), which may explain the differences in fecal BAs between the two groups. *Clostridium_scindens* likewise had a BA HSDH effect, and the other two HSDH-active bacteria, *Eggerthella* (*P* = 0.205) and *Ruminococcus_gnavus* (*P* = 0.647), both tended to be elevated in the young, but not significantly so that their overall abundance was not significantly different between the two groups (*P* = 0.769). (Figure 6D).

### Functional differences in gut microbiota between older and younger participants

To examine functional differences in gut microbiota between younger and older participants, we utilized PICRUSt 2 for predicting functional abundances and annotated functional pathways using the Kyoto Encyclopedia of Genes and Genomes (KEGG) database. Differences in functional pathways at KEGG level 2 are illustrated in Figure 6E. The elderly group displayed elevated levels in pathways related to aging, lipid metabolism, xenobiotics biodegradation and metabolism, metabolism of other amino acids, drug resistance (antimicrobial, antineoplastic), neurodegenerative diseases, cancers (specific types), and the excretory system compared to the younger group. In contrast, the younger group exhibited higher levels in cell growth and death, substance dependence, replication and repair, translation, global and overview maps, signaling molecules and interactions, transcription, and folding, sorting, and degradation.

### Association of gut microbiota with BAs and cytokines

**FIGURE 7:**
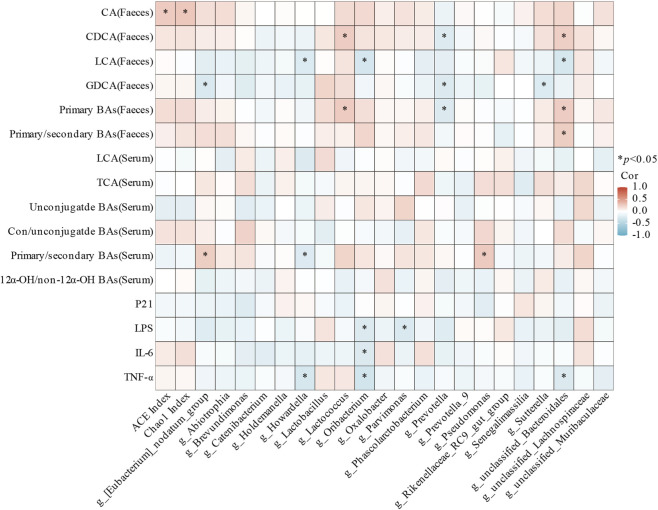
Heatmap of Spearman correlation analysis of differential gut microbiota with differential BAs and cytokines between the two groups (*P < 0.05).

We conducted Spearman correlation analyses to explore associations between changes in the BA pool and blood cytokines with variations in gut microbiota. The results are depicted in Figure 7. ACE and Chao1 indices showed positive correlations with fecal CA, indicating a higher fecal CA level in younger participants may due to abundant gut microbiota. *[Eubacterium]_nodatum_group* and *Pseudomonas* were positively correlated with primary/secondary BAs in serum. In addition, *[Eubacterium]_nodatum_group* was negatively correlated with fecal GDCA. *Howardella* showed negative correlations with fecal LCA and serum primary/secondary BAs. *Lactococcus* correlated positively with fecal CDCA and primary BAs, while *unclassified_Bacteroidales* displayed positive correlations with fecal primary BAs and negative correlations with fecal LCA. *Oribacterium* showed negative correlations with fecal LCA. *Sutterella* was negative correlations with fecal GDCA. What’s more, *Prevotella* exhibited negative correlations with fecal CDCA, GDCA and primary BAs. Notably, *Howardella, Oribacterium,* and *Parvimonas* tended to negatively correlate with LPS or inflammatory factors.

## Discussion

Numerous studies in recent years have highlighted interactions among gut microbiota, BAs, and the host may influence immune function, metabolic phenotypes, and other factors integral to the onset of various diseases (Chiang, 2013). But so far, the role of BAs and gut microbiota in aging and its specific mechanism are not yet clarified. Our study serves as a preliminary investigation into the changes and correlations between gut microbiota and BAs during the aging process.

Our study indicate a marked decrease in the ratio of primary to secondary BAs in the feces of older participants, primarily reflected in reduced primary BAs (CA and CDCA) and increased secondary BAs (LCA and GDCA). We propose that alterations in gut microbiota composition may be associated with these observed differences in fecal BAs. Notably, our gut microbiota analysis revealed a significant increase in bacteria with 7α-dehydroxylase activity in the elderly, which likely facilitates the conversion from primary to secondary BAs. Interestingly, the changes in BA concentrations in serum did not align precisely with those in feces, especially regarding the ratio of conjugated to unconjugated BAs, which exhibited an opposite trend. We hypothesize that this discrepancy may be attributed to not only the influence of gut microbiota but also to impaired BA reabsorption due to altered gut function in the elderly. BAs are primarily reabsorbed via two mechanisms (Perino et al., 2021). Most conjugated BAs are absorbed through active transport by the apical BA transporter protein (ASBT) in the terminal ileal intestinal epithelium. This is the predominant reabsorption method. Additionally, some unconjugated BAs undergo passive transport reabsorption in the small intestine and colon. As individuals age, intestinal function deteriorates, leading to weakened ASBT function at the ileum’s end and a decrease in reabsorbed conjugated BAs. Concurrently, the compromised intestinal barrier in the elderly increases the likelihood of unconjugated BAs, typically reabsorbed passively, entering the circulation. This results in a lowered ratio of conjugated to unconjugated BAs in the serum of older individuals.

Disturbances in BAs composition are significantly linked to various conditions, including obesity, diabetes, NAFLD, IBD, and various types of cancer (Cai et al., 2022; Sato et al., 2021). Although the mechanisms by which BAs influence aging remain somewhat unclear, numerous studies have highlighted their regulatory role in this process. Notably, a unique BA metabolic pathway has been identified in centenarians, potentially offering insights into longevity for those living to 100 or older (Bárcena et al., 2019). Notably, studies in long-lived populations, such as centenarians, have reported distinct bile acid signatures that may not necessarily reflect a pro-inflammatory state but instead represent adaptive host–microbiota interactions associated with healthy longevity (Sato et al., 2021). For instance, centenarians have been shown to exhibit unique bile acid metabolic profiles, potentially driven by specialized gut microbiota capable of producing beneficial secondary bile acid derivatives. In this context, the bile acid alterations observed in our elderly cohort, characterized by increased hydrophobic secondary bile acids and elevated inflammatory markers, may reflect a state of age associated dysregulation rather than adaptive remodeling. This suggests that bile acid changes during aging are heterogeneous and may represent a spectrum ranging from adaptive, health associated shifts to maladaptive alterations linked to chronic low-grade inflammation. Therefore, our findings are more likely to reflect bile acid perturbations associated with inflammaging, whereas bile acid profiles in long-lived, healthy individuals may represent a distinct, potentially protective metabolic state. Further studies directly comparing these populations are needed to clarify the differential roles of bile acid metabolism in healthy versus pathological aging.

Research also indicates that aging alters the BA pool structure in mice. Interventions like gut microbiota transplantation can modify the BA composition in aging mice, thereby extending lifespan (Ma et al., 2020) and reducing aging-related inflammation (Vancamelbeke and Vermeire, 2017). Our research found that changes in blood glucose, lipids, and blood pressure among the elderly are strongly associated with alterations in BA composition. However, these clinical and metabolic parameters themselves can influence bile acid metabolism and gut microbiota composition. In this study, analyses were primarily based on univariate comparisons and Spearman correlations, which may not fully account for potential confounding effects. Therefore, the associations observed between age and BA profiles should be interpreted cautiously, and future studies with larger cohorts and multivariable regression analyses are warranted to clarify age-specific effects. This suggests that BA irregularities may be associated with glucose and lipid metabolism disorders, contributing to age-associated diseases like hyperlipidaemia, hypertension, and diabetes mellitus.

Impaired intestinal barrier function and chronic inflammation are key aging characteristics, as observed in our elderly study participants. Elevated serum levels of lipopolysaccharide (LPS) in the elderly indicate impaired intestinal barrier function and increased bacterial endotoxin production into the bloodstream. In this study, circulating p21 was assessed as a putative marker of cellular senescence alongside established inflammatory indicators. However, it should be noted that p21 is predominantly an intracellular cell-cycle regulator, and its detection in circulation may reflect complex systemic processes rather than serving as a direct surrogate of cellular senescence. Therefore, the interpretation of serum p21 levels requires caution, and it should be considered in conjunction with well-established inflammaging markers such as IL-6 and TNF-α, rather than as an independent indicator of aging. Furthermore, our findings indicate that age-related alterations in bile acid composition, particularly the enrichment of hydrophobic secondary bile acids such as LCA, are associated with gut microbiota dysbiosis and elevated inflammatory markers. These observations support a potential interplay among microbial imbalance, bile acid remodeling, and inflammasome-related inflammatory responses, including pathways linked to NLRP3 activation. Nevertheless, given the cross-sectional design of the present study, these associations do not establish causality but instead provide a basis for future mechanistic investigations. Additionally, higher serum levels of IL-6 and TNF-α suggest increased chronic inflammation in the elderly. BAs and gut microbiota play a vital role in maintaining intestinal barrier function (Camilleri, 2019; Shi et al., 2023; Untersmayr et al., 2022). The chemical barrier, comprising mucus, digestive juices, and bacteriostatic substances secreted by the gut microbiota, forms the primary defense of the intestinal barrier (Tian et al., 2020). This barrier minimizes direct contact between the intestinal epithelium and intestinal toxins. Aging-related gut microbiota dysbiosis may impair this chemical barrier (Salazar et al., 2023). Due to their hydrophobic nature, BAs have cytotoxic effects (Schubert et al., 2017), with greater hydrophobicity increasing BA cytotoxicity (Zhao et al., 2024). Unconjugated and secondary BAs, being more hydrophobic than their conjugated and primary counterparts (Gu et al., 1992), are likelier to induce adverse effects. LCA is considered the most hydrophobic BA (Feng et al., 2022). Therefore, the increase in cytotoxic secondary BAs in the elderly may be associated with intestinal epithelial damage and impair the intestinal barrier. Moreover, certain BAs affect the proliferation and renewal of intestinal epithelial cells. LCA has been found to hinder the cell cycle and reduce the proliferative capacity of intestinal epithelial cells, exacerbating intestinal inflammation (Chen et al., 2022). Increased expression of CYP8B1, leading to a higher ratio of 12α-OH/non-12α-OH BAs, inhibits intestinal stem cell renewal and increases intestinal epithelial barrier damage by inhibiting the BA receptor (BAR) (Fiorucci et al., 2018). This aligns with our findings of elevated LCA and increased 12α-OH/non-12α-OH BA ratio in the elderly, alongside a significant correlation with elevated serum LPS levels, further confirming the critical role of BAs in intestinal barrier integrity. BAs can also directly regulate immune responses via BAR (Maruyam et al., 2002). The farnesoid X receptor (FXR) and G protein-coupled receptor (TGR5) are the primary BARs in humans (Wammers et al., 2018), highly expressed in innate immune system cells (Maruyam et al., 2002). Their activation mitigates inflammation by reducing the release of pro-inflammatory factors, such as IL-6 and TNF-α (Biagioli et al., 2017). CA and CDCA are potent endogenous FXR agonists. Their reduced levels in the elderly may weaken FXR’s inhibitory effect on inflammatory factors, contributing to chronic inflammation in this demographic.

Numerous studies have established that BAs and gut microbiota mutually regulate each other. The results of our data analysis indicate that the overall abundance of gut microbiota with 7α-dehydroxylase activity is significantly higher in the elderly. This promotes the conversion of primary BAs to secondary BAs, which precisely explains why primary BAs are lower in the feces of older adults, while secondary BAs, especially LCA, are significantly higher. Furthermore, functional validation of bile acid–transforming activities, such as BSH and 7α/β-dehydroxylase, was not performed in this study. Approaches such as *in vitro* enzyme activity assays, proteomics, or shotgun metagenomic sequencing would provide deeper mechanistic insights. Future studies incorporating these techniques are warranted to better elucidate the functional role of gut microbiota in bile acid metabolism during aging. Additionally, correlation analysis revealed that gut microbiota such as *Howardella, Oribacterium,* and *unclassified_Bacteroidales* showed negative correlation with LCA and inflammatory factors, suggesting these gut microbiota may have potentially BA biotransformation roles, might be able to promote the conversion of LCA to certain beneficial BAs and attenuate its cytotoxicity, which is worthy of further investigation. The hydrophobicity of BAs gives them antimicrobial properties (Taranto et al., 2006), which can suppress bacterial overgrowth in the intestinal tract and maintain gut microbiota balance. BARs are also key to BA gut microbiota regulation (Friedman et al., 2018). We observed that fecal CA was positively correlated with ACE and Chao1 indices, suggesting its important role in the possible maintenance of gut microbiota species richness. Primary BAs (especially CDCA) was negatively correlated with the conditionally pathogenic bacterium *Prevotella* and positively correlated with the beneficial bacterium *Lactobacillus*, which may play an important role in maintaining the balance of the gut microbiota. Notably, Prevotella has been reported to be positively associated with Th17 responses in certain pathological contexts (Zhao et al., 2023; Huang et al., 2020). However, its higher abundance in the younger group in our study may reflect context-dependent and strain-specific effects, suggesting a role in maintaining immune homeostasis rather than promoting inflammation under physiologically healthy conditions.

Our research indicates significant differences in BA profile and gut microbiota composition between elderly and young adults. These differences seem to interact, potentially influencing the aging process through glycolipid metabolism regulation, intestinal barrier function impact, and immune response modulation. In summary, the interplay between gut microbiota and BAs plays a pivotal role in aging. However, due to limited sample size and assay conditions, the full extent of their correlation may not be fully revealed, necessitating further research.

## Conclusion

This research demonstrates that aging is associated with significant alterations in BA metabolism, particularly a decline in primary fecal BAs and an increase in secondary BAs, which are associated with gut microbiota dysbiosis. These changes in BAs and gut microbiota are correlated with key biomarkers of metabolic dysfunction, including blood pressure, glucose, lipid levels, and liver function markers, as well as markers of chronic low-grade inflammation such as P21, LPS, IL-6, and TNF-α. These findings highlight the complex interplay between gut microbiota and BA metabolism in aging, suggesting that disrupted BA and microbiota homeostasis may be involved in intestinal barrier dysfunction and systemic inflammation. However, due to the cross-sectional design, causal relationships cannot be established.

## Data Availability

The raw data supporting the conclusions of this article will be made available by the authors, without undue reservation.
