# Supplementary material for: The alteration of bile acids and gut microbiota is associated with intestinal barrier dysfunction and inflammaging in human
Source: Front Aging. 2026 Apr 15;7:1741360. doi: 10.3389/fragi.2026.1741360 (PMC13124955; doi:10.3389/fragi.2026.1741360)
Supplement: Supplementary file 3 [file Table1.docx]

**Table S1-1.** Results of differential analysis of fecal bile acids between the two groups.

| Bile acids(ng/mL) | Young(n=100) | Old(n=100) | *P* value |
| --- | --- | --- | --- |
| CA | 286.500(45.0,2003.8) | 1070.000(235.0,2760.0) | 0.002** |
| CDCA | 364.000(163.5,1232.5) | 1250.000(372.8,3525.0) | 0.000** |
| UDCA | 794.000(156.3,2157.5) | 1050.000(302.0,2485.0) | 0.175 |
| HDCA | 306.000(75.5,1816.0) | 289.500(50.1,1161.0) | 0.216 |
| DCA | 2340.500(1427.5,5315.0) | 2165.000(956.3,3975.0) | 0.332 |
| LCA | 1130.000(675.8,3642.5) | 839.000(434.8,2102.5) | 0.034* |
| TCA | 32.000(9.4,113.0) | 50.800(13.8,129.0) | 0.362 |
| TCDCA | 49.700(20.6,148.5) | 51.400(14.1,132.8) | 0.504 |
| GCA | 51.600(28.8,113.3) | 49.400(26.8,119.0) | 0.897 |
| GCDCA | 86.850(27.9,204.3) | 88.400(34.7,163.0) | 0.968 |
| TUDCA | 4.550(2.1,10.3) | 4.750(2.4,9.4) | 0.851 |
| THDCA | 0.728(0.3,2.3) | 1.180(0.4,2.7) | 0.221 |
| TDCA | 6.050(1.5,16.2) | 9.590(3.5,23.8) | 0.062 |
| TLCA | 312.000(166.0,616.0) | 457.000(198.0,646.0) | 0.119 |
| GUDCA | 9.180(4.0,25.3) | 9.200(4.2,16.7) | 0.669 |
| GDCA | 17.950(7.3,49.2) | 12.900(2.6,28.7) | 0.020* |
| GLCA | 11.400(4.9,19.0) | 11.450(5.4,22.6) | 0.716 |
| Conjugated BAs | 1048.799(452.9,1504.9) | 970.270(629.3,1464.9) | 0.824 |
| Unconjugated BAs | 7540.000(4252.8,18259.0) | 10038.050(6107.9,17060.8) | 0.163 |
| Primary BAs | 1777.295(464.5,4609.0) | 3053.735(1315.7,7919.7) | 0.001** |
| Secondary BAs | 5531.028(3633.3,16581.6) | 5873.215(3718.1,10839.4) | 0.951 |
| 12α-OH BAs | 3937.345(2318.5,8417.3) | 4054.815(2456.5,7024.6) | 0.959 |
| non-12α-OH BAs | 4848.445(2598.4,11841.1) | 6733.005(3824.8,11366.5) | 0.177 |
| Total BAs | 9256.095(5277.7,19913.9) | 10968.159(6775.4,18169.4) | 0.203 |

Ratios of different classes of fecal BAs

| Ratio | Young(n=100) | Old(n=100) | *p* value |
| --- | --- | --- | --- |
| Conjugated/unconjugated BAs | 0.097(0.0,0.2) | 0.089(0.0,0.2) | 0.607 |
| Primary/secondary BAs | 0.230(0.1,0.8) | 0.460(0.1,1.0) | 0.007** |
| 12α-OH/non-12α-OH BAs | 0.710(0.5,1.0) | 0.712(0.4,1.0) | 0.267 |

**Table S1-2.** Results of differential analysis of serum BAs between the two groups.

| Bile acids(ng/mL) | Young(n=100) | Old(n=100) | *P* value |
| --- | --- | --- | --- |
| CA | 77.350(23.5,160.8) | 44.300(18.0,111.5) | 0.121 |
| CDCA | 196.500(86.2,435.8) | 189.500(79.5,386.3) | 0.709 |
| UDCA | 52.950(22.7,88.0) | 45.400(20.7,68.9) | 0.146 |
| HDCA | 1.360(0.4,2.8) | 1.100(0.4,1.7) | 0.162 |
| DCA | 148.000(64.9,225.0) | 125.000(46.2,178.5) | 0.173 |
| LCA | 5.570(1.6,14.2) | 3.140(1.6,5.7) | 0.006** |
| TCA | 8.460(3.6,23.9) | 17.000(6.0,38.3) | 0.008** |
| TCDCA | 26.550(11.5,70.8) | 49.500(16.9,84.1) | 0.054 |
| GCA | 62.000(27.6,134.5) | 67.300(37.6,134.0) | 0.246 |
| GCDCA | 299.000(167.0,582.0) | 377.000(219.0,824.0) | 0.11 |
| TUDCA | 0.871(0.4,3.2) | 1.275(0.6,3.4) | 0.157 |
| TDCA | 6.855(3.1,11.6) | 8.410(3.2,18.7) | 0.167 |
| TLCA | 77.200(65.1,95.0) | 84.200(60.1,104.0) | 0.223 |
| GUDCA | 28.000(17.1,67.7) | 36.100(15.1,64.8) | 0.963 |
| GDCA | 41.400(17.4,85.5) | 46.850(18.9,82.1) | 0.775 |
| GLCA | 2.180(0.6,4.8) | 1.750(0.7,3.3) | 0.226 |
| Conjugated BAs | 652.582(395.5,1178.4) | 756.095(478.4,1357.9) | 0.161 |
| Unconjugated BAs | 489.510(279.6,926.3) | 382.970(234.4,735.1) | 0.033* |
| Primary BAs | 918.360(489.2,1558.9) | 985.650(566.4,1675.8) | 0.464 |
| Secondary BAs | 393.506(259.9,622.2) | 345.975(221.0,510.7) | 0.107 |
| 12α-OH BAs | 367.295(238.6,580.1) | 315.730(190.9,533.0) | 0.12 |
| non-12α-OH BAs | 970.039(559.0,1449.6) | 987.577(618.7,1621.6) | 0.468 |
| Total BAs | 1389.914(837.8,2155.7) | 1322.011(808.3,2076.2) | 0.984 |

Ratios of different classes of serum BAs

| Ratio | Young(n=100) | Old(n=100) | *p* value |
| --- | --- | --- | --- |
| Conjugated/unconjugated BAs | 1.178(0.6,2.5) | 1.863(0.9,3.8) | 0.011* |
| Primary/secondary BAs | 2.181(1.3,3.5) | 2.845(1.8,4.2) | 0.043* |
| 12α-OH/non-12α-OH BAs | 0.425(0.3,0.7) | 0.387(0.2,0.5) | 0.031* |

Data are expressed either as mean ± SD or median (25th, 75th percentiles), depending on whether they follow a normal distribution. Differences in means were analyzed using independent samples t test, and differences in medians were analyzed using Wilcoxon rank sum test. Significant p values are marked (*p <0.05, **p <0.001).
